# Foreign Language Training to Stimulate Cognitive Functions †

**DOI:** 10.3390/brainsci11101315

**Published:** 2021-10-03

**Authors:** Sol Herrera Naranjo, Mercedes Suárez de la Torre, Francia Restrepo de Mejía, David Facal

**Affiliations:** 1 EDUTLAN Research Group, Department of Foreign Languages, Faculty of Education and Human Sciences, Universidad de Córdoba, Montería 230002, Colombia; solherrera@correo.unicordoba.edu.co; 2 CITERM Research Group, Coordinator of the PhD. Program in Cognitive Sciences, Faculty of Social and Business Studies, Universidad Autónoma de Manizales, Manizales 170006, Colombia; mercedessuarez@autonoma.edu.co (M.S.d.l.T.); francia46@gmail.com (F.R.d.M.); 3 NEUROAPRENDIZAJE Research Group, Neurophysiology Laboratory, Universidad Autónoma de Manizales, Manizales 8811177, Colombia; 4 Department of Developmental and Educational Psychology, Universidad Santiago de Compostela, 15782 Santiago de Compostela, Spain

**Keywords:** cognitive training, foreign language training, tip-of-the-tongue, lexical access, working memory, processing speed

## Abstract

Adult development throughout a lifetime implies a series of changes in systems, including cognitive and linguistic functioning. The aim of this article is to study the effect of foreign language training on linguistic processing, particularly the frequency of the tip-of-the-tongue (TOT) phenomenon and on other cognitive processes such as processing speed and working memory in adults aged 40 to 60 years. Sixty-six healthy Colombian teachers were enrolled in this study. They were then randomly divided into an experimental group (33 healthy adults who underwent a four-week training period) and a passive control group (33 healthy adults who did not undergo any training). All participants performed induction tasks for the TOT phenomenon, working memory and processing speed before and after the four weeks. Results showed more of an effect in the semantic access, phonological access and processing speed measures with a better performance in the experimental group than in the control group. In Colombia, this type of training is still new and little is known to date about programs to prevent cognitive impairments. The need to conduct more studies confirming or refuting these findings is discussed, thus raising awareness about the extent of this type of training to increase the linguistic and cognitive performance of adults.

## 1. Introduction

Adult development implies a series of changes in the human systems, including cognitive and linguistic functioning changes. There is evidence of changes in cognitive abilities after the age of 30, with a certain decline in fluid abilities such as working memory or processing speed [1,2,3,4,5], and language skills such as lexical access (particularly the tip-of-the-tongue (TOT) phenomenon), contrary to the vocabulary knowledge that is preserved throughout the years [6,7,8,9,10]. These changes are reflected in a general slowing down of processes, alteration of working memory and different changes in language comprehension and production [7,11,12,13].

Research advancements in the field have aimed at developing relevant strategies to foster cognitive functioning against a possible cognitive decline in the late stages of human life [14,15]. Thus, different cognitive training approaches have been proposed and shown to be effective in increasing cognitive functioning [16,17,18,19]. Empirical evidence in the training of domain-specific tasks in the participants’ mother tongues, such as linguistic tasks, has proven to improve verbal working memory and processing speed which are important cognitive processes in oral production processes and are related to lexical access difficulties [20,21,22,23,24,25]. Similarly, in recent years, research has advanced in intensive foreign language training and its effects on cognition. Findings on this type of training, in which specific foreign language aspects are implemented in a learning process, have showed changes in the brain structure of regions related to the mother tongue, in cognitive functions and in linguistic performance of the language used [26,27,28].

There is recent and relevant literature on intensive foreign language learning programs, in which target language learning is achieved in general [15,29,30,31,32,33]. Klimova conducted a review on the effects of foreign language learning among older adults, highlighting potential positive effects in three areas: enhancement of cognitive functions, self-esteem and increased opportunities for socializing [15]. Ware et al. found that a technology-based English training program for older French adults was feasible for this population and that the participants found it stimulating and enjoyable, although differences in feelings of loneliness and social isolation did not change after the 16-week, 2-h weekly sessions training program [29]. Kliesch et al. found an improvement in older German adults after three weeks of 60 English lessons. In this pilot, intensive study of the participants with higher verbal fluency and working memory capacities showed faster learning than those with lower verbal and cognitive capacities [30]. Valis et al. conducted an intervention through English language teaching and learning for 12 weeks (three 45-min lessons per week) with 20 older Czech adults, and only obtained a slight enhancement of the cognitive status of the participants using the MoCA test [31]. From a qualitative perspective, these researchers found that foreign language learning with peers of the same age is beneficial for the individual’s social and psychological well-being, developing social networks and gaining self-confidence in learning capabilities. Training in a foreign language would also promote changes in the central nervous system, i.e., changes in the brain’s network involved with the inferior frontal gyrus. In addition, language training foments a more resistant connectivity in the left supramarginal gyrus, which is associated with the phonological form storage. The gray matter structure would also change in the hippocampus and the effects on cognitive processing would be shown [34,35,36,37,38,39,40,41]. Bellander et al. [34] trained young adults with a foreign language vocabulary (Italian) for 10 weeks and found changes in the gray matter structure in the hippocampus, in addition to a relationship between the episodic memory and the training performed. These authors found that the performance in a delayed matching-to-sample task was a significant predictor of the neural changes. In the task, images of three-dimensional colored objects were briefly shown and then the object was presented again mixed with four highly similar objects to be recognized. Hosoda et al. [36] reported a similar finding after intensively training a group of young, non-English speaking individuals with specific English vocabulary for 16 weeks and observed a significant increase in the gray matter volume in the lower right frontal gyrus. Furthermore, the researchers found an increase in connectivity in specific pathways that support foreign language learning, such as network formations of the inferior frontal gyrus with the caudate nucleus that supports semantic lexical control when learning a foreign language [42], and high connectivity in the temporal part of the arcuate fascicle related to phonological processing. These results also coincide with Wong et al. [35] and Veroude et al. [26] who observed neural changes associated with intensive foreign language vocabulary training in adults. These were basically changes in a network involved with the inferior frontal gyrus and a stronger connectivity in the left supramarginal gyrus related to phonological form storage. Likewise, Mårtensson et al. [39] proved that after intensive foreign language learning in adulthood, there were changes in the brain’s region structures associated with language. Schlegel et al. [40] also reported a similar finding after an intensive, nine-month foreign language course. These authors’ research showed significant changes in the tracts of the frontal lobe, specifically in the corpus callosum which is an area generally excluded in current neural models of language processing. Alternatively, Qi et al. [38] demonstrated an association between the white matter structure and the successful learning of phonology with an intensive four-week course. More recently, with the purpose of observing cognitive functioning in healthy seniors, studies have coincided in reporting that cognitive performance can be promoted with foreign language learning [29,31,32].

The characteristics of these training approaches are still heterogeneous with respect to their duration, intensity, methodology, sample size and even the outcome assessment measures [27,43,44,45], which have made understanding the impact of this type of training difficult. However, findings show that most of these training approaches are promising and that a systematically organized approach with foreign language study can be effective in the target population of this study. To date, no empirical evidence has been found in Colombia or Latin America of foreign language interventions carried out in middle-aged people who are aging normally. Therefore, due to the limited research in this field, the aim of this article is to study the effects of foreign language training in English on lexical access in Colombian adults aged 40 to 60 years whose native language is Spanish, thus observing the effect of this training on the frequency of the TOT phenomenon, working memory performance and processing speed. In other words, the purpose was to carry out training that involved specific language aspects such as vocabulary and phonology, and then match with English as a facilitator, which has not been done to date in the target group. 

## 2. Materials and Methods

### 2.1. Participants

Sixty-six volunteers, a non-probabilistic sample due to the commitment for the entire training and accessibility, were enrolled in this study. They were primary and secondary education teachers from the urban area of the public sector of the city of Montería (Colombia), randomly distributed into an experimental group and a control group. The experimental group with n = 33 participants had an average age of 55.4 (SD = 6.2). This group was 70% female and 30% male. Their average educational level was 5.93 years (SD = 0.69) of post-secondary education. The control group with n = 33 participants had an average age of 52.5 (SD = 6.2). This group was 63.33% female and 36.67% male. Their average educational level was 6.17 years (SD = 1.02) of post-secondary education. The control group was thus marked as a passive group, i.e., without any foreign language training or other specific activity. None of the participants reported neurological, sensory or psychiatric disorders, or linguistic pathologies, nor did they consume substances that affected their performance of tasks. None of the participants were teachers of English as a foreign language. All participants provided their informed consent, following the Declaration of Helsinki principles. The study protocol was approved by the Bioethics Committee of the Universidad Autónoma de Manizales (Universidad Autónoma de Manizales Bioethics Committee, minute 76, 2018). Finally, both groups, experimental and control, were composed of 30 participants due to a lack of time and overlap in working schedules. It is noteworthy that, *a priori*, three participants were added to each group in order to guard against potential data loss [46,47].

### 2.2. TOT Task

A TOT elicitation task was used. This task was designed using 200 low-frequency Spanish words taken from the Spanish Frequency Dictionary [48]. These words were randomly divided with 100 words applied in the pre-test and 100 words in the post-test following these morphosyntactic categories: 25 object names, 25 verbs, 25 adjectives and 25 abstract names [7]. In addition, only Spanish words that were phonologically matched to the foreign language [English] in the initial syllable were taken into account (e.g., balcón-balcony, misterio-mystery). This was consistent with empirical studies that have reported a positive effect on target retrieval by using phonological priming such as the first syllable [49,50] and the cross-linguistic influence of a foreign language reported on different studies [51,52,53]. Additionally, the task words were selected from an initial pool of 300 words that allowed the elimination of those words that were ambiguous or whose definitions were circular. Spanish definitions were taken from the *Diccionario de la Real Academia Española RAE* [54] and the *Diccionario de Uso del español de América y España* VOX [55].

Following the experimental procedure developed to experimentally assess TOTs [7,56,57], after presenting the definition of each word the participants had to name the target word corresponding to the given definition. If the participants named the word correctly, it was registered and they continued with the next word; in the same way, if they did not know the right word, that would also be registered and they would then continue with the next word. If they did not name the word but claimed to know it and did not remember it, it was recorded as a TOT. Then, they were asked if they remembered the first syllable. During this state, if the subject did not spontaneously remember the word, a recognition test was performed [7]. Here, the participant was presented with three options of words, including the target word. If the subjects named the right word, they continued with the next word and it was counted as a positive TOT. After the presentation of each word, a familiarity test was carried out with the purpose of determining the degree of familiarity of the words presented to the subjects [56]. The categories presented were well known, known, fairly known, few known and unknown. The results of this test showed that more than 90% were concentrated in the “well known” category.

Semantic access (SA) was calculated with the formula: [Knows + positive TOTs/Total words] defined by Juncos-Rabadán et al. [56] based on the method proposed by Gollan and Brown [57]. This refers to the proportion of SA responses (correct responses and positive TOTs) over the total number of words. The phonological access (PA) was calculated with the equation: [Knows/Knows + positive TOTs] which represents the proportion of the recovery of the word form of all the cases in which the semantic recovery has occurred [56].

### 2.3. Working Memory and Processing Speed Tasks

Participants underwent two working memory subtests (retention of digits and numbers and letters) and two processing speed subtests (search for symbols and keys), both taken from the Wechsler Memory Scale IV [58] in order to evaluate their performance on working memory and processing speed, respectively.

### 2.4. Cognitive and Linguistic Training with English as a Foreign Language

**Figure 1:**
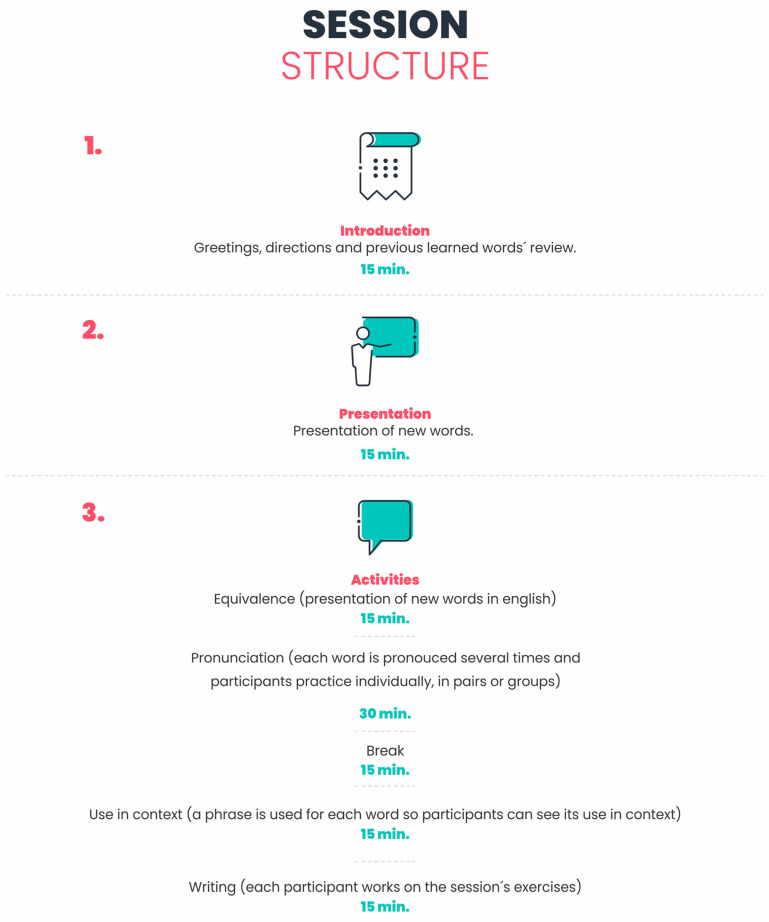
Structuring of each session.

The training applied to the experimental group was carried out for a four-week period with an intensity of 10 h per week, distributed in eight contact hours and two hours per week of independent work. This training was named ToTEFL (Tip of the Tongue in English as a Foreign Language) [59] and the general objective of the training was to develop, through practice in English as a foreign language, a better linguistic–cognitive performance. This would allow the participating subjects to access their Spanish lexicon more easily (specifically when they experienced a state of TOT), increase the fluent cognitive resources and through these improvements have more effective oral communication in the mother tongue/first language [Spanish]. This training was designed specifically for this study, including specific components of English as a foreign language (vocabulary, phonetics and phonology). Throughout the training the team worked with the Spanish words selected for the TOT task (half of the words were phonologically matched with the foreign language [English] and the other half were not). In addition, an image was assigned to each word, the correspondence in English to its respective phonetic transcription for each word and a context constructed from a statement or sentence. With these elements, daily activities were designed that included linguistic exercises. These activities included different exercises on verbal fluency, naming, vocabulary exercises with phonetic or semantic aids, word recognition and word or sound association exercises, word ordering and completing sentences using words or images, among others (see Figure 1). The training application was organized based on the morphosyntactic categorization proposed by Burke et al. [7]. In the first week, the words corresponding to the object names category were practiced; in the second, words categorized as verbs; in the third, words in the adjective’s category; and in the fourth, the words corresponding to the abstract names’ category. Additionally, a follow-up was carried out with individual exercises at home (weekend) for two hours a week, and also a weekly vocabulary test to verify the learning of the total words trained weekly (50 words).

### 2.5. Statistical Analysis

The averages of both the pre-test and the post-test were compared using the Student’s t-test for the data that were normally distributed. The Wilcoxon signed rank test was used for those data that were not normally distributed. To carry out the analysis of the interactions between groups (experimental and control) and between time (pre-test and post-test), a two-way ANOVA with repeated measures was performed. The independent variable included an intra-subject time factor (pre-test and post-test) and an inter-subject group factor (experimental and control). The dependent variables were SA and PA in a TOT task, working memory measured through retention of digits and numbers and letters, and processing speed measured through search for symbols and keys’ tasks. The level of significance for all tests was *p* < 0.05. The IBM SPSS Statistics Version 24.0 (Armonk, NY, USA) was used for all analyses.

## 3. Results

Table 1 shows the results of SA, PA, working memory and processing speed between the pre-test and the post-test in both the experimental group and the control group. With respect to the experimental group, there were significant changes between the pre-test and the post-test (*p* < 0.05) and the average differences of the results between the pre-test and the post-test were the following: for the TOT test, the mean difference for SA was 0.10 and for PA it was −0.40. With regard to working memory, for the digit retention subtest the average difference was −3.13 and in the number and letter sequence subtest it was −3. For processing speed, the mean difference for the symbol search subtest was −3.13 and for the keys subtest it was −5.10. In general, the results show that there was an improvement in the results of the variables evaluated in the experimental group after training with a foreign language between the pre-test and the post-test. Alternatively, the participants in the control group presented changes between the pre-test and the post-test; however, the average difference of the results was lower than that of the experimental group. This could be observed in the results of all the variables. For SA, the average difference was −0.10 and for PA, −0.04. With respect to working memory, for the digit retention subtest the average difference was −1.73 and in the number and letter sequence subtest it was −2.43. For the processing speed variable, the mean difference for the symbol search subtest was −2.56 and for the keys subtest it was −3.83.

Through the two-way ANOVA, it was verified that there was a significant difference in the intra-subject variables (pre-test and post-test) in the results of all the dependent variables, which means that the effect of the time factor was significant. For the TOT variable, a significant difference was found for both indicators (SA and PA). For PA, the results were: F (1, 116) = 259.11 and *p* < 0.05. For SA, the results were: F (1, 116) = 14.90 and *p* < 0.05. For the working memory variable, the results for digit retention were: F (1, 116) = 16.39 and *p* < 0.05. For a sequence of numbers and letters the results were: F (1, 116) = 32.88 and *p* < 0.05. Finally, for the processing speed variable, the results for symbol search were: F (1, 116) = 5.02 and *p* < 0.05 and for keys, F (1, 116) = 4.01 and *p* < 0.05. 

The inter-subject effect test (experimental and control) indicated that there was a significant difference in the results of both indicators of the TOT variable (SA and PA). The results for PA were: F (1, 116) = 167.29 and *p* < 0.05. For SA, the results were: F (1, 116) = 103.89 and *p* < 0.05. For the working memory variable, the results were not significant for retention of digits (F (1, 116) = 0.69) nor for succession of numbers and letters (F (1, 116) = 0.15). For the processing speed variable, the results showed that there was a significant difference for symbol search: F (1, 116) = 4.90 and *p* < 0.05. For the key subtest this difference was not significant: (F (1, 116) = 3.54. This means that the effect of the group factor is only significant for the lexical access measures and for the processing speed measure, in the symbol search test. Finally, the results for the interaction group*time were significant for the PA variable: F (1, 116) = 168.63, *p* ≤ 0.05, and for the SA variable: F (1, 116) = 71.70, *p* ≤ 0.05.

## 4. Discussion

As could be established from the results, an effect of ToTEFL training on lexical access was found. This effect could be verified after observing the results of the analysis of the PA and SA indicators, resulting from the transformation of the TOT frequency scores. While the improvement in SA is to be expected given the increased exposure to linguistic stimuli through the training situations, the improvement in PA is relevant given the presence of lexical access difficulties such as the TOT phenomenon throughout adult development. According to the Transmission Deficit Hypothesis (TDH) [7], receiving training in a foreign language [English] would improve the transmission of activation from the semantic nodes to the phonological form in the mother tongue (Spanish) as well, improving lexical access. On the other hand, despite the fact that in this study the size of the differences between the pre- and post-test for the PA and SA variables was zero or close to zero, the size of these differences in the measures is due to the transformation required by the indirect measures of the TOT phenomenon, obtaining significantly higher results in the post-tests of the experimental group compared to those of the control group for both measures (PA and SA). These results are in line with the findings of Facal et al. [60] who calculated PA and SA measures in the same way and obtained a similar effect size in two successive measures, baseline and follow-up; however, in their case it was a longitudinal study and not an experimental intervention study. 

This study did not aim for the people in the experimental group to learn the foreign language in extent as well as in complexity, as could be observed in studies of foreign language learning [29,30,31,32,33,38,39,40,41]. Complementary to these, the results of this research focusing on language training with a foreign language show that, with specific intensity and specific language exercises in vocabulary, phonology and foreign language equivalents, changes in linguistic processing such as lexical access, specifically the TOT phenomenon, are observed. In this sense, the language training approach is more specific than that of a broader foreign language acquisition process and could have more short-term effectiveness on specific linguistic and cognitive processes [27]. Among the strategies proposed to solve TOT conditions, in addition to priming, theoretical proposals for interventions that would solve the TOT condition through linguistic and/or cognitive training are known [27,43,44,45]. This study provides empirical evidence of training with a foreign language to observe its effects on linguistic processing and thus demonstrate another way to solve the difficulties in accessing the lexicon.

In addition to showing changes in linguistic processing, the training carried out in this study also showed positive changes in processing speed. It should be noted that, although the two processing speed tests are closely related and there is evidence that they measure the same construct [58], in this research more effectiveness was found in the symbol search test. It is important to mention that processing speed begins its decline before the age of 50 years and occurs in people with a generalized decline in cognitive functioning which can cause cognitive difficulty at early ages [4,10,61]. The results obtained indicate that people who participated in language training improved their concentration, their ability to perform in a state of time pressure and their visuo-perceptual discrimination. Meta-analyses conducted by Reijnders et al. [19] showed that there are previous cognitive training approaches that improved the processing speed with healthy adults. This coincided with the results of the review conducted by Tardif and Simard [17] and the study carried out by Ball et al. [62] with a short training of five to six weeks, that also showed improvements in processing speed. Our ToTEFL training also achieved improvements in performance on one of the processing speed tests, despite not being a training style specifically designed to improve these abilities.

Alternatively, in the present study no inter-subject effect was found between the experimental and control groups in working memory. There are cognitive training approaches that showed immediate increases in verbal working memory scores with the use of linguistic exercises in the mother tongue [20,21,23,24,63,64]. Bellander et al. [34] conducted intensive training with a foreign language in a healthy adult population and did obtain a significant effect size in memory processes other than working memory. In this regard, it is worth noting that Facal et al. [65], through a structural equation model in which the performance of a TOT task, vocabulary tests, processing speed tests and working memory tests were related, found relationships between the variables measured in the TOT task and the results of the processing speed tests, but not between the TOT variables and the results of the working memory tests. The authors explain that, assuming that TOT events involve controlled processing, the relationship between processing speed and TOT across the lifespan could be interpreted as reflecting changes in voluntary compensatory mechanisms. However, processing resources measured across working memory tasks would better reflect working memory–vocabulary relationships, which would be less dependent on temporal limits in processing.

It should be noted that in Colombia, as far as is known, this type of training is still quite unknown and, in general, campaigns for early detection and prevention of cognitive decline are still lacking. Therefore, ToTEFL training can improve semantic access, phonological access and processing speed, and therefore might be an intervention able to prevent cognitive decline and promote cognitive health. Similarly, the type of activities, intensity and methodology of the ToTEFL training were an essential contribution to preventing the difficulties of cognitive decline explicitly manifested in language (TOT phenomenon), in middle-aged, fully healthy individuals. The limitations of this study include the repetition of the working memory and processing speed tests, so it is important that in subsequent studies the learning effects that could arise from this situation be controlled. In addition, other limitations include the lack of registration for bilingual participants speaking languages other than Spanish or English, the fact that the control group was entirely passive and no alternative interventions were designed and the lack of evidence for improvements over longer periods due to the short follow-up time. All these limitations can be properly considered as subjects for future research. It is also recommended that future research carry out studies of a comprehensive scope to deepen the motivational and affective aspects involved in learning a foreign language in adult samples trying to improve their cognitive functioning. In addition to this, the authors also suggest the development of studies on the relationship between TOT and processing speed in greater depth.

## 5. Conclusions

The ToTEFL linguistic-cognitive training had an effect on linguistic processing in both SA and PA. It was also found that once the training was completed, those who participated in the training increased their performance in processing speed. Linguistic training in a foreign language could be a successful strategy to prevent cognitive decline in Spanish-speaking adults.

## Figures and Tables

**Table 1:** Measures of central tendency and dispersion for each variable. TOT: tip-of-tongue phenomenon, PA: phonological access, SA: semantic access, WM: working memory, PS: processing speed, DR: digit retention, SNL: succession of numbers and letters, SS: symbol search, KEYS: keys subtest.

VARIABLE		EXPERIMENTAL		CONTROL	
		Average	Standard Deviation	Average	Standard Deviation
TOT	PA PRE	0.42	0.11	0.42	0.07
	PA POST	0.84	0.05	0.46	0.07
TOT	SA PRE	0.95	0.06	0.93	0.06
	SA POST	1.00	0.00	0.78	0.09
WM	DR PRE	18.40	2.50	19.60	3.22
	DR POST	21.53	3.07	21.33	4.16
WM	SNL PRE	14.87	2.96	15.33	2.70
	SNL POST	17.87	2.06	17.77	2.58
PS	SS PRE	18.73	7.34	21.83	7.61
	SS POST	21.87	7.66	24.40	4.88
PS	KEYS PRE	45.93	10.19	50.77	10.61
	KEYS POST	51.03	14.38	54.60	13.18

## Data Availability

The data presented in this study are available on request from the first author.
